# Robotic-Assisted Thoracoscopic Resection of the First Rib for Vascular Thoracic Outlet Syndrome: The New Gold Standard of Treatment?

**DOI:** 10.3390/jcm10173952

**Published:** 2021-08-31

**Authors:** Adrian Zehnder, Jon Lutz, Patrick Dorn, Fabrizio Minervini, Peter Kestenholz, Hans Gelpke, Ralph A. Schmid, Gregor J. Kocher

**Affiliations:** 1 Department of Surgery, Cantonal Hospital of Winterthur, 8401 Winterthur, Switzerland; adrian.zehnder@ksw.ch (A.Z.); hans.gelpke@ksw.ch (H.G.); 2 Division of General Thoracic Surgery, Bern University Hospital, University of Bern, 3010 Bern, Switzerland; jon.lutz@insel.ch (J.L.); Patrick.Dorn@insel.ch (P.D.); ralph.schmid@insel.ch (R.A.S.); 3 Department of Thoracic Surgery, Kantonsspital Luzern, 6004 Lucerne, Switzerland; fabrizio.minervini@luks.ch (F.M.); peter.kestenholz@luks.ch (P.K.)

**Keywords:** robotic, first rib resection, thoracic outlet syndrome, minimal-invasive

## Abstract

In thoracic outlet syndrome (TOS) the narrowing between bony and muscular structures in the region of the thoracic outlet/inlet results in compression of the neurovascular bundle to the upper extremity. Venous compression, resulting in TOS (vTOS) is much more common than a stenosis of the subclavian artery (aTOS) with or without an aneurysm. Traditional open surgical approaches to remove the first rib usually lack good exposure of the entire rib and the neurovascular bundle. Between January 2015 and July 2021, 24 consecutive first rib resections for venous or arterial TOS were performed in 23 patients at our institutions. For our completely portal approach we used two 8mm working ports and one 12/8 mm camera port. Preoperatively, pressurized catheter-based thrombolysis (AngioJet^®^) was successfully performed in 13 patients with vTOS. Operative time ranged from 71–270 min (median 128.5 min, SD +/− 43.2 min) with no related complications. The chest tube was removed on Day 1 in all patients and the hospital stay after surgery ranged from 1 to 7 days (median 2 days, SD +/− 2.1 days). Stent grafting was performed 5–35 days (mean 14.8 days, SD +/− 11.1) postoperatively in 6 patients. The robotic approach to first rib resection described here allows perfect exposure of the entire rib as well as the neurovascular bundle and is one of the least invasive surgical approaches to date. It helps improve patient outcomes by reducing perioperative morbidity and is a procedure that can be easily adopted by trained robotic thoracic surgeons. In particular, patients with a/vTOS may benefit from careful and meticulous preparation and removal of scar tissue around the vessels.

## 1. Introduction

Thoracic outlet syndrome (TOS) involves three different structures that may contribute to the symptomatology to varying degrees. Anatomically, the subclavian vein lies most anteriorly and is bounded by the subclavius muscle anteriorly and by the anterior scalene muscle posteriorly. The anterior and middle scalene muscles encompass the subclavian artery and the brachial plexus. They insert on the postero-lateral aspect of the first rib. Some patients also suffer from congenital bands and fibrous slips interdigitating with neurovascular structures. This fact elucidates why mechanical compression is the principal cause of symptoms [1]. We differentiate between neurogenic, non-specific, venous and arterial TOS.

**Figure 1:**
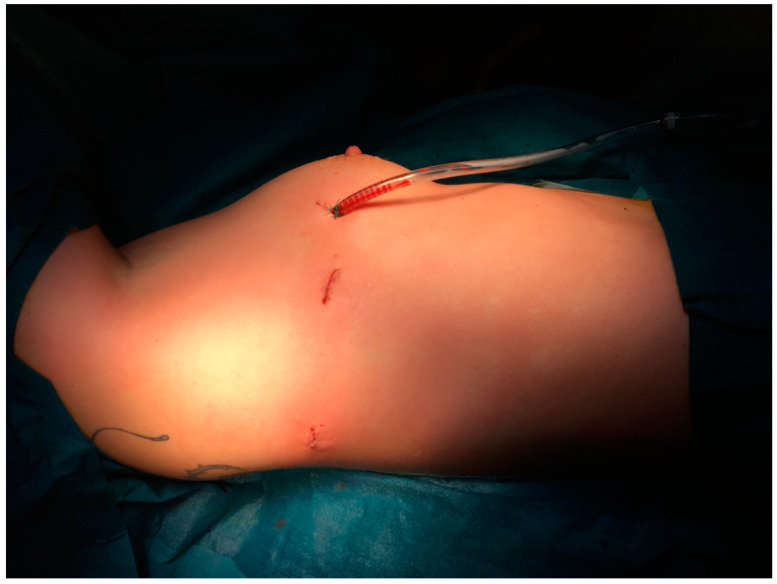
Completely portal approach using only three incisions; extraction through ventral port (drain indwelling).

As described in our recent report, the mainstay of treatment for vascular TOS or in cases refractory to conservative treatment is surgery [2]. All known approaches to date primarily lack good exposure or overview of the entire first rib and do not provide easy accessibility because of straight endoscopic instruments. Thus, they carry the risk of injury to the neurovascular bundle due to strong traction and inadequate exposure or the risk of recurrence due to incomplete resection. For this reason, in 2015, we began using our newly developed robotic-assisted technique, which has the advantage of overcoming these restraints and providing perfect exposure of the entire rib, including evidence of the final result [2]. Soon we refined the technique, performing this operation with only three incisions, which has not been described in the literature before (Figure 1).

## 2. Methods

Between January 2015 and July 2021, 24 consecutive first rib resections were performed in 23 patients at our institutions. The patient cohort consisted of 7 men and 16 women. Informed consent was obtained from all patients prior to study inclusion. The median age was 32.5 years (range 15 to 73 years), and resections were performed on the left side in 6 patients and on the right side in 18 patients, and bilaterally (in a staged fashion) in 1 patient.

The distribution of etiologies is shown in Table 1.

The usual diagnostic steps consisted of physical examination with Adson- and Wright tests. Magnetic resonance imaging in deliberate provocation of the upper extremities proved to be crucial also for non-specific or neurogenic TOS (to exclude a vascular component in the latter). In addition, we performed neurophysiological evaluation with senso-motoric neurography and needle myography.

In all vascular entities, patients presented with arm swelling, pain, or heaviness. The work-up consisted of duplex ultrasonography and angiography. At the same time, catheter-based thrombolysis (AngioJet^®^) could be performed if possible and indicated.

All patients underwent routine follow-up at 3 months, 6 months and 1 year postoperatively. In addition, all vascular TOS patients were evaluated on a regular basis by angiologists using duplex-sonography for patency of vessels after 3 months and 12 months.

### Surgical Technique

Under general anesthesia, a double-lumen tube was placed, and patients were placed in a lateral decubitus position. Under one-lung ventilation, a 12 mm camera trocar was placed in the fifth intercostal space in the midaxillary line, and after insertion of the 30° angled camera, CO_2_ insufflation was started at 8 mmHg. Two 8-mm working trocars were then placed in the third intercostal space in the anterior axillary line the other one just below/behind the tip of the scapula, respectively. The correspondent intercostal spaces were infiltrated posteriorly under visual control with a local anesthetic (a total of 20 mL of 0.75% ropivacaine). The Si and Xi version of the daVinci robot was used for all operations and the patient cart was positioned over the head of the patient, and the dissection of the first rib was started using a Cadiere grasper on the left and a bipolar Maryland forceps on the right arm. First the pleura over the first rib is opened and then the intercostal muscles are separated from the rib. Medially to the subclavian vein the rib is then divided by means of a Kerrison rongeur, which is introduced through the posterior 8mm trocar. Once the rib is divided, the medial rib end can be pushed down, creating a trap door mechanism that allows perfect visualization and dissection of the ligaments and scalene muscles, that attach to the first rib. Once the scalene muscles are divided, the neurovascular bundle is completely freed and slightly drops into the thoracic cavity as a sign of complete decompression.

In the posterior part, the rib is then cut few millimeters lateral to the passage of the T1 nerve root over the rib, also with a Kerrison rongeur inserted through the medial robotic trocar. The entire specimen is then retrieved, the lung re-inflated, and a chest tube placed.

The following link provides a short video of our technique:

https://vimeo.com/589229914/ac54c4f123 (access on 18 August 2021)

https://youtu.be/om5q2UV2RrI (access on 18 August 2021)

## 3. Results

Complete resection of the first rib was achieved in all patients [2]. Operative time ranged between 71 and 219 min (median 117 min, SD +/− 35.7 min). The following hospital stay ranged from 1 to 4 days (median 2 days, SD +/− 2.1 days). Apart from a postoperative hematoma at the level of the neck in one patient who underwent an additional cervical rib resection, no intra- or postoperative adverse events occurred, and no additional thoracic intervention was required. Since we used thrombolysis via pressure catheter in the preoperative phase of vTOS, this procedure led to acute renal failure in one case (patient aged 32 years), which resulted in a delay of the surgery by four weeks until renal function was fully restored.

The median pain score on the first postoperative day was 3 on a visual analog scale (range 0–7) in the perioperative period. The usual treatment consisted of the following medications: Paracetamol, NSAID or metamizole. In patients with venous TOS and recent thrombosis, the therapeutic treatment with oral or subcutaneous anticoagulants was initiated on the first postoperative day and routinely continued up to 3 months postoperatively, depending on whether impaired venous return or residual thrombus was detected on the postoperative venous duplex scan [3,4].

In 6 of 17 patients a stent grafting was performed 1–5 weeks after surgery due to residual and relevant stenosis caused by scar tissue around the vein (35.3%).

At follow-up, patients showed complete (n = 18) or partial (n = 6) relief of symptoms. In two patients, the partial relief was due to a traumatic etiology of symptoms. Three patients underwent surgery on both sides because symptoms on both sides were almost identical and treatment on the first side was very successful.

We found only one recurrent thrombosis due to residual thrombus in the postoperative period 3 months after surgery. Re-stenting using pull-through technique was performed in our angiology department. In this case, compliance with oral anticoagulants was a problem. The patient was working under heavy physical stress and could not follow our instructions for a consequent anticoagulant therapy.

Two patients were treated with venous stent grafts prior to surgery before being referred to our institution. The stent was inserted 10 months and 4 weeks before surgery, respectively, by interventional radiologists who attempted to reopen the vessel and overcome the stenotic section. This approach was unsuccessful because the mechanical compression was not resolved, and the stent graft was left with stenosis. Moreover, the foreign body is thrombogenic and re-thrombosis occurs soon after the intervention. Surgery in such cases is also more challenging because scar tissue complicates dissection around the neurovascular bundle. Nevertheless, no complications occurred in this difficult situation thanks to the perfect overview and precision.

In the first patient, the initial phase was un-eventful, and the stent remained in place, but with a relevant stenosis. After one year, a venous bypass was implanted by the vascular surgeons. The second patient underwent prolonged stent-in-stent placement by interventional radiologists during the same hospital stay with the result of good inflow at the end.

## 4. Discussion

Initially, we operated mainly on neurogenic or non-specific TOS patients in whom symptoms persisted despite physiotherapeutic treatment. Then, referrals from our angiologists and vascular surgeons increased very rapidly as they too recognized the benefit of the robotic minimally invasive first rib resection technique for our patients. The various etiologies are listed in Table 1. In addition to five arterial TOS, the nineteen other vascular patients suffered from recurrent thrombosis of the subclavian vein (79.2%).

### 4.1. Operative Procedure

The surgical procedure for TOS patients has been described in different techniques. There are open, video-assisted and robotic-assisted procedures. Most of them have specific advantages but also some limitations that need to be considered for a good clinical long-term outcome [2,5,6,7,8,9,10,11,12,13,14,15].

In the era before the development of video-assisted technologies, a transaxillary or supra-/infraclavicular approach was usually chosen. These approaches provide a direct view of only certain parts of the rib. Depending on whether a vascular or neurologic etiology is suspected, one can selectively dissect either the medial or posterior portion of the rib. Especially in cases of venous TOS, some authors prefer an infraclavicular incision because it provides excellent exposure of the subclavian vein and allows direct reconstruction if needed [4,14]. However, some risk to the brachial plexus and inadequate exposure of the posterior portion of the first rib may complicate this procedure. In neurogenic TOS, reconstructive surgeons prefer the supraclavicular approach to achieve a thorough neurolysis of the brachial plexus. An incomplete resection of the anterior portion of the first rib as well as the costoclavicular ligament and the subclavius tendon are quite common when using that technique. In addition to these specific approaches, a transaxillary incision has been widely used in thoracic surgery for various etiologies since it has been first described in 1966 [16,17]. Unlike VATS procedures, in which a chest drain is placed in most cases to monitor potential intrathoracic bleeding for 24 h, transaxillary or sub./infraclavicular approaches usually require no chest tube. Some surgeons might argue, therefore, that postoperative pain might be an issue having no interference with subcostal nerves. Since we routinely use local anaesthetics for all incisions with a small caliber chest tube (20 charrier) and quick removal on postoperative day one this point is neglectable in our experience. The VAS (visual analog scale) for pain on the first postoperative day does hardly exceed the mean value of 3 (+/−2.01) at rest. There is also no need to raise the arm/shoulder in a vertical fashion which might cause postoperative discomfort due to a continuous stress on the brachial plexus and the shoulder muscles [16].

Since the first steps in video-assisted thoracic surgery in the early 1990s of the last century, this minimally-invasive method has become widely accepted for almost every kind of intrathoracic procedure [18]. In addition, for the first rib resection it has been brought up as a technique with benefits over the open approach. Namely, this includes an increased view along the full length of the rib with precise identification of all the neurovascular bundle. It also avoids injury to the intercosto-brachial cutaneous nerve as there is no need of a 6–10 cm incision along the thoracic wall [12]. Even the patient’s obesity is an issue since a more demanding procedure plays hardly any role in this approach.

In 2012, Gharagozloo et al. described the first completely robotic-assisted technique for thoracic outlet syndrome, mainly as a treatment of Paget-Schroetter disease. This newly developed approach consisted of 4 incisions and helped to overcome some of the restraints of conventional VATS-surgery.

Of course, it is difficult to show the true benefit of the RATS approach to other minimally-invasive approaches as we already know from lung cancer surgery. The differences are quite small and George et al. 2017 showed also very good results with low morbidity in 10 VATS procedures [12]. On the other hand, the robot enables the surgeon to easily dissect very narrow spaces, around the corner of the rib in perfect visibility of the delicate structures. The ergonomic advantages for surgeons have been proven in other fields of surgery [19].

With the robotic system, we are able to improve limited maneuverability of the conventional videoendoscopic instruments with endo-wrist movement and enhance visualization by 3D vision and high-definition magnification [8,15]. Other authors present similar results with the same technique [20,21,22]. Our distinguished goal was to improve this technique by reducing the ports needed for resection of the first rib. In fact, we now perform the whole procedure with only three incisions, the biggest no larger than 1.5 cm for extraction of the rib. To avoid an additional retractor or a utility incision, CO2-insufflation is used to create a working space without the danger of affecting the non-ventilated lung. In fact, a three-port approach is also described by Wybaillie et al. in 2018, Yogeswaran et al. in 2020, and Hoexum et al. in 2021 [13,23,24]. Since the first author presents a case report of a cervical rib and the latter two resect only part of the first rib (also one case report), we truly believe that our aforementioned technique stands alone in its proper way and extent. Since Mingoli et al. 1995 (and also George et al., 2017) showed clearly an inferior outcome due to a long posterior stump, we are confident that less than a complete resection will not ameliorate the clinical results.

Therefore, this presents a novel technique for the benefit of the patients thanks to the possibilities of a new technology that might justify some higher costs of the system. Taking into account the referenced literature in the text, adoption of the approach by different surgeons is spreading worldwide these days and the costs of robotic systems are coming down.

Three cases of arterial TOS were found to have an additional cervical rib, which had to be removed in the same procedure and through the same three port incisions. Because two of these patients had aneurysms of the artery, this vessel had to be replaced with an autologous vein graft from the great saphenous vein. This part of the procedure was performed through a separate infraclavicular incision with perfect postoperative results during follow-up.

### 4.2. Paget-Schroetter Disease

In recent years, we have treated more and more patients with vascular impairment, which is why the collaboration with vascular surgeons and angiologists as well as interventional radiologists is very important.

In venous TOS, external venous compression can often cause primary thrombosis which is also known related to a trauma in a restrictive thoracic outlet. It is named as Paget-Schroetter syndrome or “thrombose par effort” and related to excessive upper extremity exercises or exertion. The term Paget-Schroetter syndrome has been introduced by Hughes in 1949 who reviewed 320 cases of upper-extremity in the medical literature [25]. However, the syndrome of thrombosis involving the subclavian vein has been firstly described by Paget (London) in 1875 and Von Schroetter (Vienna) in 1884 [26,27].

Males and laborers performing strenuous work are predominantly concerned whereas women suffer from neurogenic TOS to a greater extent. The right upper extremity is affected more often presumably because of the prevalence of dexterity in mankind irrespective of the etiology of TOS [1]. In our series, the number of right sided interventions reached as much as 19 cases (73%).

A very rare entity of TOS corresponds with the so-called subclavius posticus muscle. These fibres originate from the sternal part of the first rib and insert at the superior border of the scapula. They run along the anterior surface of the subclavian vein and then cross the artery and brachial plexus as well. MRI diagnostics may reveal such anatomical variant. Therefore, the muscle should also be taken into consideration when TOS symptoms are present [28,29].

Of course, also a secondary thrombosis due to intimal injury by a foreign body is quite common these days. It is usually associated with the presence of central lines, pacemakers, pulmonary artery catheters or other invasive monitoring. The alteration of vascular structures results in a localized area of increased thrombogenicity [30]. Therefore, so far, we could not definitively assign a specific case to this entity.

### 4.3. Treatment Timeframe

Concerning the timeframe between treatment of acute thrombosis and the operative intervention of first rib resection no clear guidelines exist. Like in current practice, we use a pressurized catheter-based thrombolysis (AngioJet^®^) to resolve an acute occlusion of the subclavian vein. According to published case series the procedure should be scheduled at least 2–4 weeks after the intervention when the associated thrombosis is resolved [15,31]. Madden et al. even performed first rib resection in the same hospitalization [4]. We followed these principles and planned surgery 2–4 weeks after the intervention whereas in one case the operation has been delayed for more than 2 months due to patients wishes. The rational thought behind the timeframe is a resolution of perivenous inflammation associated with the effort thrombosis event. No repeated CT scans, angiography or duplex ultrasounds were needed in the absence of related clinical findings. Patients were continuously put on oral anticoagulant drugs (OAC) until the operation, which were very well tolerated.

Follow-up of vein patency mainly consisted of duplex ultrasound or angiography after about four to six weeks with or without placement of stents or balloon angioplasty. The angiological follow-up examination by means of clinical evaluation and imaging studies took place at three or six months, as well as one year, postoperatively. Beside one difficult case which included a lack of the patient’s compliance concerning OACs, all examination showed good patency of the subclavian vein. There is no clear guideline if and how long OAC treatment should be administered. Most of the authors state a duration of three to six months when a stent is in place. In absence of residual thrombus as well as absence of stent placement it might be permissible to omit OAC.

## 5. Conclusions

The minimally invasive robotic approach to first rib resection described here is safe and reproducible and results in improved patient outcomes compared with traditional open techniques. It, therefore, has the potential to become the new state-of-the-art technique in this field.

## Figures and Tables

**Table 1:** Patients with vTOS had predominantly the right side affected.

Type of TOS	No. of Procedures	Left	Right
Venous TOS	19	4	15
Arterial TOS	5	2	3
Total	24	6	18
